# MicroRNA-487a-3p inhibits the growth and invasiveness of oral squamous cell carcinoma by targeting PPM1A

**DOI:** 10.1080/21655979.2021.1884396

**Published:** 2021-03-16

**Authors:** Lishan Wang, Shuqing Ge, Futing Zhou

**Affiliations:** Department of Maxillofacial Surgery, Weifang City People’s Hospital, Weifang City, Shandong Province, China

**Keywords:** Oral squamous cell carcinoma, OSCC, protein phosphatase 1A, PPM1A, miR-487a-3p

## Abstract

Oral squamous cell carcinoma (OSCC) forms the majority of the entire cancerous tumors which occur in the mouth. Current treatment advances, such as surgical resection, chemotherapy, and radiotherapy, have significantly helped reduce OSCC. However, the overall patient survival rate remains relatively low. MiRNAs, a non-coding RNA group, are essential for multiple biological functions, which are essential for the progression of cancer, including survival of the cell, migration, multiplication, differentiation, and apoptosis. The study aimed to explore the existing association between miR-487a-3p and PPM1A and elucidating their role in modulation of proliferation in OSCC cell lines. In this study, we used CAL-27 and TCA-8113 OSCC cell lines and human samples to validate our results. The manifestation of miR-487a-3p and PPM1A was checked using quantitative real-time PCR. The miR-487a-3p and PPM1A binding was investigated through western blot assay and dual-luciferase reporter gene. Functional experiments, including colony formation, CCK-8, and transwell experimentations, were undertaken to validate cells’ growth and invasion activities. According to the results, the expression of miR-487a-3p is regulated in the OSCC cell lines compared to normal cells. Moreover, the mimicking of miR-487a-3p significantly reduces the OSCC cell growth and invasion, and PPM1A overexpression exerts oncogenic effects and hinders the anti-oncogenic effects of miR-487a-3p. In conclusion, the study demonstrated that miR-487a-3p might act as a tumor suppressor by inhibiting the growth and invasion of OSCC via regulating PPM1A expression.

## Graphical Abstract

**Figure:**
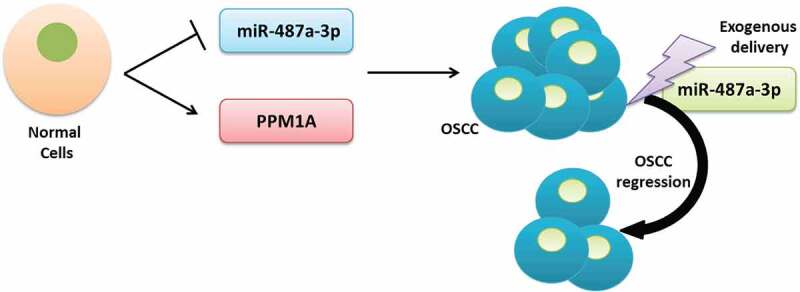

## 1. Introduction

Oral squamous cell carcinoma (OSCC) constitutes about 90% of the entire tumors in the mouth [1]. According to reports, 275,000 new OSCC incidences are reported annually [2]. Increased cases are linked to alcohol consumption, smoking, inadequate oral hygiene, and prolonged malnutrition [3]

Clinically, painless hard or soft tissue wounds, ulcers or lamps, mouth sores, and difficulty during tongue movement are the early OSCC indications [1]. Although OSCC has significantly been managed through various approaches, including surgical resection, chemotherapy, and radiotherapy [4], patients’ survival rate only remains 66% [5]. Further, the complications of surgery affect swallowing and speech [6]. Consequently, alternative approaches such as research elucidating the internal tumorigenesis mechanism are essential for potential molecular-targeted therapy.

MicroRNAs (miRNAs) are small non-coding RNA particles of 19–23 nucleotides [7]. According to reports, miRNAs are significant in gene expression, regulation, and cell signaling transduction [8]. MiRNAs engage in multiple steps which are critical for cancer progression, including cell survival, proliferation, specialization, migration, and apoptosis [9–11]. Further, miRNAs have been linked with the tumorigenesis of OSCC. MiR-494, for instance, worsens OSCC by aiming Bmi1 and ADAM10 [12]. miR-506 is also crucial for suppression and invasion of OSCC cells via GATA6 [13]. Furthermore, miR-133 plays a tumor suppressor role in various metastatic cancers, where it inhibits invasion migration of cells via FOXC1 target in pituitary adenoma. Abnormal miR-487a-3p regulation has been linked with several tumors, such as breast cancer, hepatocellular carcinoma, and various activities that promote cancer progression. Nevertheless, no study has reported a relationship between miR-487a-3p and OSCC.

Protein phosphatase Mg2+/Mn2+ dependent 1A (PPM1A) is a protein phosphatase 2 C (PP2C) family molecule, which regulates MAPK (c-jun- N-terminal kinase/p38) Cdk6 and Cdk2, tumor growth factor-β/Smad [14–16]. PPM1A also controls nerve growth factor-activated Akt/ERK [17], tumor proliferation [18], migration, and cell invasion [19].

The present study hypothesized the essential involvement of miR-487a-3p in the suppression of OSCC via PPM1A targeting. Further, the investigation aimed to determine the effect of expression/overexpression of miR-487a-3p on OSCC, to determine whether PPM1A is a target protein of miR-487a-3p in OSCC, and to determine the effect of PPM1A Knockdown in OSCC. We reported the involvement of miR-487a-3p during OSCC growth and inhibition of invasion through PPM1A suppression.

## 2. Materials and methods

### 2.1 Tissue samples

Our institutional Ethics Review Committee approved the study. A prior written informed consent was obtained from all the study patients. Surgically excised OSCC tissues were acquired from 20 patients who had tested positive for OSCC and went through surgical resection at our hospital during March 2017-July 2019. None of the OSCC patients had undergone a chemotherapeutic, radiotherapeutics, or any specialized therapy before surgery. Besides, matching adjacent non-diseased tissues were acquired from 20 patients in the same hospital. All the experimental specimens were quickly snap-frozen and kept at −150°C for future use.

### 2.2 Cell culture

Cell lines were acquired from the Chinese Academy of Sciences cell bank. CAL-27 and TCA8113 cells were cultured in Dulbecco’s modified Eagle’s medium DMEM, while HOK cells were grown in Oral keratinocyte medium, supplemented with 10% fetal bovine serum at 37°C and 5% CO_2_. Cells were divided when 70–80% confluence had been achieved. Cells rinsing and digestion was done using PBS and 0.25% trypsin, respectively. Finally, the cell suspension was centrifuged for 5 minutes at 1000 rpm and inoculated into new culture plates.

### 2.3 Cell transfection

CAL-27 and TCA8113 experimental cells were randomly divided into various groups then transfected using miR-487a-3p mimics, PPM1A-siRNA, PPM1A-OE, and the comparable negative controls (miR-NC, siRNA-NC, and pcDNA), respectively. Oligonucleotides and plasmids were synthesized from Dharmacon, China. Each group contained approximately 3 × 10^5^ cells. The cells were transfected using Lipofectamine 2000^TM^ (Invitrogen, Carlsbad, CA, USA). Summarily, cells (of 60–70% confluence) were seeded in 6-well plates and transfected with the required oligonucleotides and/or plasmids.

### 2.4 Extraction of RNA and RT-qPCR

cDNAs were synthesized through RNA reverse transcription. RT-qPCR was used to determine the miR-487a-3p and PPM1A expressions. The assay conditions were established as follows: a) pre-degeneration at 95°C in 10 minutes, b) 95°C degeneration for 30 seconds, c) 60°C annealing for 30 seconds, and finally, 72°C extensions for 30 seconds. Relative miRNA and mRNA expression was determined using the 2^−∆∆*Ct*^ equation. U6 and β-actin were the miRNA and mRNA controls, respectively. Primers used in this study are: U6-F 5’ CTCGCTTCGGCAGCACA; U6-R 5’ CGCTTCACGAATTTGCGT; miR-487a-3p-F 5’AGCCGGTCCAGTACACCTTT; miR-487a-3p-R 5’-GGAAAGCACCGTCTGTTGTT; PPM1A-F 5’ TGGCGTGTTGAAATGGAG; PPM1A-R 5’ AGCGGATTACTTGGTTTGTG; β-actin-F 5’ GCACCACACCTTCTACAATG; β-actin-R 5’ TGCTTGCTGATCCACATCTG.

### 2.5 CCK-8 assay

The changes during cell propagation were assessed by Cell Counting Kit-8 (CCK-8) (Dojindo, Japan). Summarily, approximately 5 × 10^4^ cells/mL were seeded in every well of a 12-well plate, and 10 µl of CCK-8 was added to each well, 48 h post-transfection. The optical cell density (OD) was finally determined in a microplate reader at 450 nm.

### 2.6 Dual-luciferase reporter gene experiment

Wild-type and mutant PPM1A-reporter vector construction were done using PGL3-promoter vectors (Promega, Madison, USA). The oligonucleotide sequences for luciferase assay were as follows: PPM1A-WT 5’CCAGCCAAUUUUUGUUGUAUGAUU; PPM1A-MUT 5’ CCAGCCAAUUUUUGUUGAUGCCUU, miR-NC or miR-mimics, each at 20 nM, were transfected using either 3’UTR-wt or 3’UTR-mut in TCA8113 and CAL-27 cell lines. The cell luciferase activities were assessed 48 h later with a dual-luciferase reporter system (Promega, Madison, USA).

### 2.7 Transwell assay

Cell invasion assay was done using inserts of eight pores, pre-coated with Matrigel. . After 48 h of transfection, cells were harvested and re-suspended in DMEM medium without serum. A suspension of1 × 105 cells per mL was then grown in the upper chambers. Complete DMEM medium supplemented with 10% FBS was the only constituent of the lower compartment. Cells invasion was observed after 24 hours, then the non-invading cells in the upper compartment were removed using a cotton wool swab. Cells that had finally settled on the lower membrane were fixed in 4% paraformaldehyde, and later stained in 0.05% crystal violet. Random microscopic observation of at least four fields was done, and cells quantified using NIH-ImageJ software.

### 2.8 Colony formation assay

Approximately 2 × 10^2^ cells/well were inoculated on six-well plates for two weeks after transfection. Cell fixation and staining were then done in 10% formaldehyde and 0.1% crystal violet dye, respectively, for 15 minutes. The stained colonies were finally counted in a microscope.

### 2.9 Extraction of protein and western blot

Total and cellular protein was extracted using RIPA lysis buffer with protease inhibitor cocktail. Cells were then centrifuged at 4°C for 20 minutes, 15,000 g. Protein concentration was subsequently determined by Bradford assay. The resulting sample lysates were then loaded on 10% (w/v) Tris-HCl sodium dodecyl sulfate-polyacrylamide gel electrophoresis gels (SDS-PAGE) and set to run on a 100 V power. The samples were later changed to polyvinylidene fluoride membrane (PVDF) (Millipore). Blocking of PVDF was then done for 1 hr in 5% skimmed milk, and later incubated using anti- PPM1A rabbit monoclonal antibody, a rabbit monoclonal antibody against β-actin (internal control) overnight at 4°C. The membranes were then washed three times in Tris-buffered saline-Tween 20 (TBS-T) and later incubated using an anti-rabbit antibody. Finally, bands were pictured using Immobilon^TM^ HRP substrate (Millipore).

### 2.10 Statistical analysis

Data were analyzed using SPSS 21.0. The data measurement was introduced as Mean ± SD. The difference between experimental sets was compared using one-way ANOVA or Student’s t-test. The difference were significant if *P < 0.05 and **P < 0.01.

## 3. Results

Theoretically, miRNAs have been linked with the regulation of multiple steps critical for cancer progression, including cell survival, proliferation, specialization, migration, and leading to apoptosis. The study aimed to explore the existing association between miR-487a-3p and PPM1A and elucidating their role in modulation of proliferation in OSCC cell lines. In this study, we used OSCC cell lines and human samples to validate our results. CAL-27 and TCA8113 OSCC cell lines were transfected with miR-487a-3p mimics, PPM1A-siRNA, or PPM1A-OE. The manifestation of miR-487a-3p and PPM1A was determined using quantitative real-time PCR. The miR-487a-3p and PPM1A binding was investigated by western blot assay and dual-luciferase reporter gene. Functional experiments, including colony formation, CCK-8, and transwell experimentations, were undertaken to validate cells’ growth and invasion activities.

### 3.1 Expression of miR-487a-3p is downregulated during oral squamous cell carcinoma

**Figure 1.**
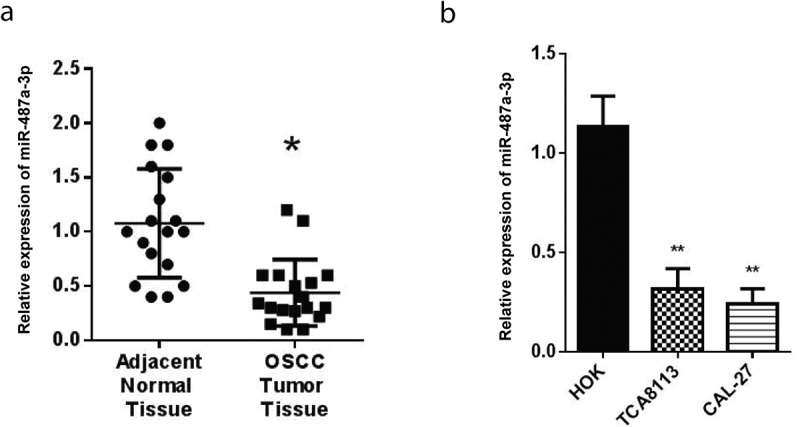
Expression of miR-487a-3p is downregulated during oral squamous cell carcinoma

RT-qPCR was used to detect the miR-487a-3p expression in OSCC tumors and adjacent OSCC tissues. According to our findings, in parallel with adjacent normal tissues, miR-487a-3p was significantly reduced in OSCC tumors (Figure 1(a)). We carried out RT-qPCR to determine the expression of miR-487a-3p in CAL-27, HOK, and TCA8113 cells. The outcomes showed significantly reduced miR-487a-3p in CAL-27 and TCA8113 OSCC cell lines than HOK normal cells (Figure 1(b)).

(a) Expression of miR-487a-3p in OSCC tissue. (b) Expression of miR-487a-3p in OSCC cell lines TCA8113 and CAL-27. *P < 0.05 and **P < 0.01.

### 3.2 *The miR-487a-3p overexpression inhibits growth and invasion of oral squamous cell carcinoma* in vitro

**Figure 2.**
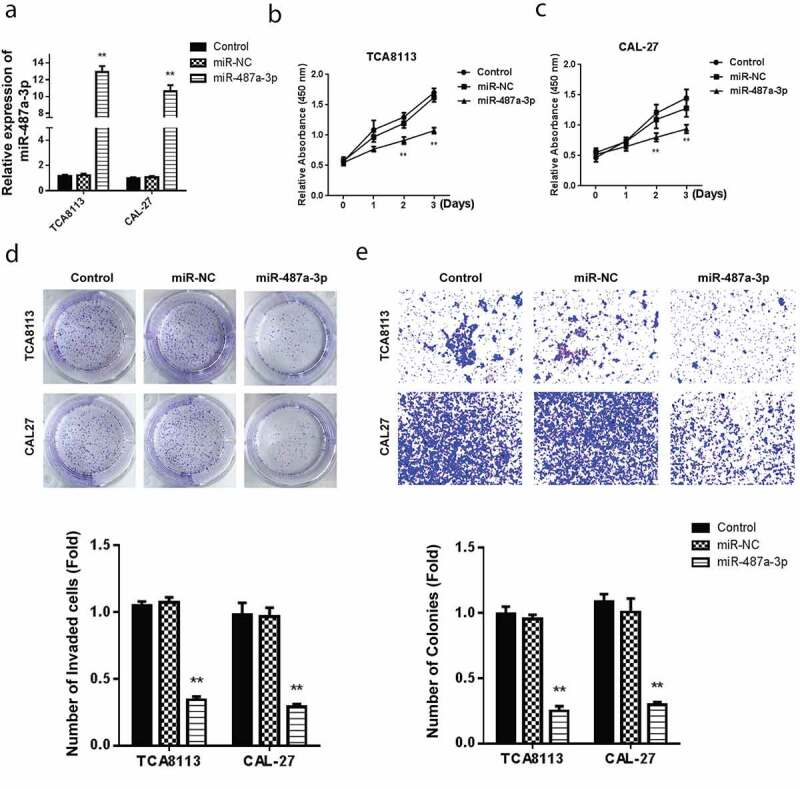
Overexpression of miR-487a-3p impedes cell growth and invasion in OSCC *in vitro.*

Assessment of miR-487a-3p mimics in OSCC cell lines confirmed a significantly higher miR-487a-3p in both TCA8113 and CAL-27 OSCC cell lines, as demonstrated in Figure 2(a). OSCC cell lines treatment with miR-487a-3p mimics and viability assessment using the CCK-8 assay demonstrated a significantly reducedTCA8113 and CAL-27 cells’ (Figure 2(b,c)). The colony formation assay reported a significant reduction in the cell growth following transfection with miR-487a-3p mimics, as shown in Figure 2(d). Using the transwell assay in TCA8113 and CAL-27 cells, transfection of miR-487a-3p mimics lead to a significant cell invasion reduction (Figure 2(e)). The results showed an overall miR-487a-3p suppression in the OSCC. Mimicking miR-487a-3p can also significantly reduce the cell growth and cell invasion in OSCC.

(a) Assessment of the miR-487a-3p mimics transfection in OSCC cell lines. (b,c) CCK-8 analysis with miR-487a-3p mimics or NC in OSCC cell lines. (d)Colony formation analysis for cell growth with miR-487a-3p mimics or NC in OSCC cell lines. (e) Transwell invasion analysis for cell invasion with miR-487a-3p mimics or NC in OSCC cell lines. *P < 0.05 and **P < 0.01.

### 3.3 PPM1A is a target protein of miR-487a-3p in oral squamous cell carcinoma (OSCC)

**Figure 3.**
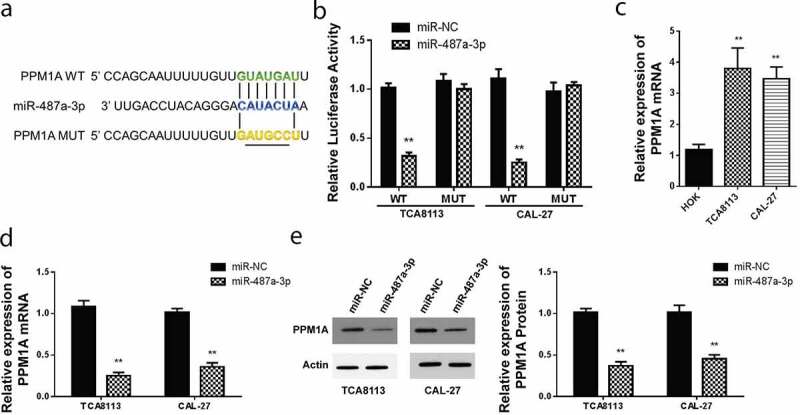
Targeting of PPM1A by miR-487a-3p in OSCC cells

Theoretically, PPM1A is Smad phosphatase, which is essential in the dephosphorylation and promotion of TGFβ-activated Smad2/3. PPMIA expression has been confirmed to abolish the transcriptional and anti-proliferative effects induced by TGFβ, hence its overall oncogenic role (Lin et al., 2006). Analysis of miR-487a-3p target gene, using targetscan miRNA target prediction tool reported that PPM1A has a site which it uses to bind miR-487a-3p in the 3’UTR region shown, as shown in Figure 3(a). For verification, the dual-Luciferase reporter experiment was used to detect luciferase activity. The miR-487a-3p, mutant, and wild type PPM1A 3’UTR was illustrated in Figure 3(a). PPM1A wt or Mut 3’UTR in CAL-27 luciferase analysis in CAL-27, and TCA8113 cells post-transfected with miR-487a-3p mimics indicated a significantly reduced luciferase activity in CAL-27 and TCA8113 cells transfected using WT PPM1A and miR-487a-3p mimics (Figure 3(b)). However, miR-487a-3p did not represent any change in the relative luciferase activity when cells were transfected using the MUT PPM1A plasmid. We further used RT-qPCR to evaluate the PPM1A mRNA expression in both cell lines, and we found up-regulated levels in both OSCC cell lines, i.e., CAL-27 and TCA8113, as compared to the normal HOK cell line (Figure 3(c)).

(a) 3’UTR region of PPM1A containing the binding site for miR-487a-3p in WT and MUT plasmids. (b) Luciferase activity of PPM1A WT or MUT 3’-UTR with miR-487a-3p mimics or NC in OSCC cell lines. (c) The expression of PPM1A mRNA is up-regulated in OSCC cell lines (TCA8113 and CAL-27). (d) The expression of PPM1A mRNA affected with miR-487a-3p mimics or NC in OSCC cell lines. (e) The expression of PPM1A protein affected with miR-487a-3p mimics or NC in OSCC cell lines. *P < 0.05 and **P < 0.01.

Further analysis of PPM1A mRNA expression was done for CAL-27 and TCA8113 transfected with miR-487a-3p mimics. In this case, PPM1A mRNA levels were significantly repressed following miR-487a-3p mimics transfection in both CAL-27 and TCA8113 cells compared to miR-NC mimic (Figure 3(d)). Finally, we used western blot assay to study PPM1A protein expression in CAL-27, and TCA8113 miR-487a-3p mimics transfected cells. We observed significant downregulation of PPM1A in cells transfected using mimics of miR-487a-3p, but not in the cells transfected using miR-NC in both the OSCC cell lines (Figure 3(e)). These data suggested that miR-487a-3p binds to the PPM1A 3’UTR and regulate its expression at the mRNA and protein level.

### 3.4 PPM1A knockdown represses oral squamous cell carcinoma growth and invasion

**Figure 4.**
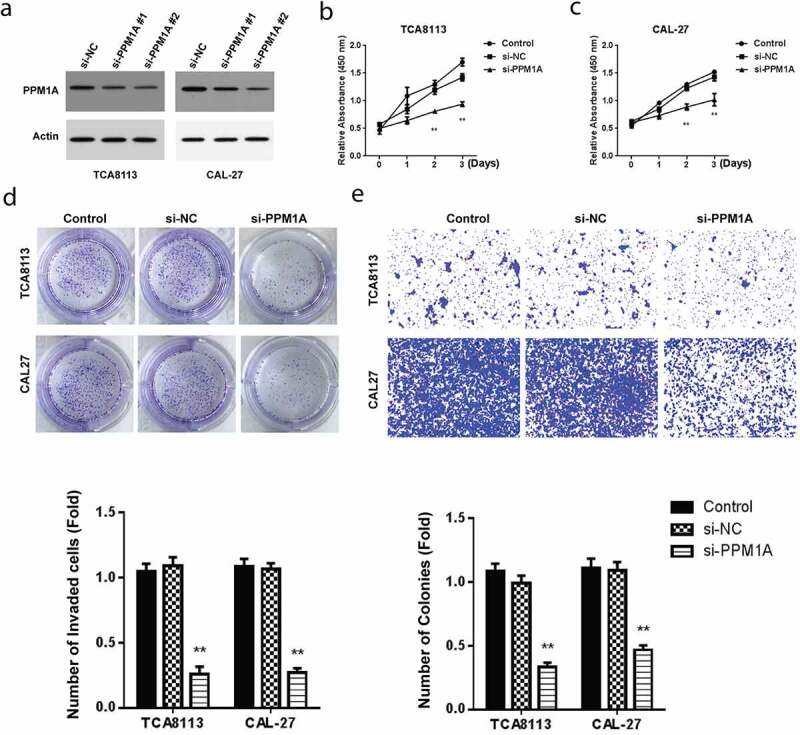
Knockdown of PPM1A suppresses OSCC growth, and invasion

To study the effect of PPM1A on OSCC, CAL-27, and TCA8113 cells were transfected using various PPM1A siRNAs and then PPM1A protein expression was determined using western blot. As demonstrated in Figure 4(a), the PPM1A expression reduced in TCA8113 and CAL-27 cells transfected with si-PPM1A. We then evaluated cell proliferation by CCK-8 assay in CAL-27 and TCA8113 cells post-transfected using si-PPM1A or si-NC. Our findings showed a significantly reduced proliferation activity after transfection with si-PPM1A (Figure 4(b,c)).

(a) Expression of PPM1A protein affected by si-PPM1A siRNAs in OSCC cells. (b,c) CCK-8 analysis with si-PPM1A or NC in OSCC cell lines. (d) Colony formation analysis for cell growth with si-PPM1A or NC in OSCC cell lines. (e) Transwell invasion analysis for cell invasion with si-PPM1A or NC in OSCC cell lines. *P < 0.05 and **P < 0.01.

Further, cell growth analysis in TCA8113 and CAL-27 cells post-transfected with miR-487a-3p mimics or NC hinted at a significant drop in cell growth activity after transfection with si-PPM1A (Figure 4(d)). Finally, the transwell invasion technique was used to evaluate cell invasion in TCA8113 and CAL-27 cells post-transfected with miR-487a-3p mimics or miR-NC. Cell invasion activity was significantly decreased after transfection with si-PPM1A (Figure 4(e)). Overall, these results demonstrated the involvement of PPM1A in the growth and invasion of OSCC cells.

### 3.5 Mimicking miR-487a-3p exerts therapeutic effects by alleviating cell growth and invasion to rescue the oncogenic effects of PPM1A in oral squamous cell carcinoma cells

**Figure 5.**
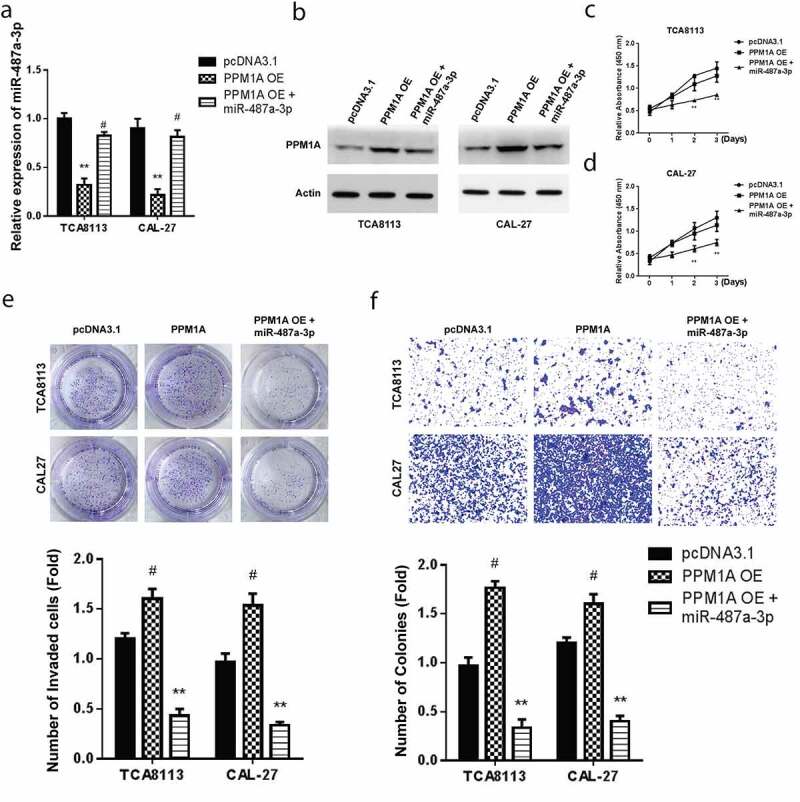
Mimicking miR-487a-3p rescues the oncogenic effects of PPM1A in OSCC

Transfection of CAL-27 and TCA8113 cells with pcDNA-PPM1A overexpression (OE) or combination of miR-487a-3p and PPM1A OE plasmid was done to clarify the effects of miR-487a-3p and PPM1A in OSCC. The findings demonstrated significantly reduced miR-487a-3p levels in TCA8113 and CAL-27 transfected with PPM1A OE plasmid and in the cells co-transfected using miR-487a-3p and pcDNA-PPM1A OE plasmid (Figure 5(a)). Western blot assay was then used to assess PPM1A protein levels in the individual cells co-transfected with a control plasmid, PPM1A OE, and miR-487a-3p + PPM1A OE. PPM1A was significantly increased in both OSCC cells transfected using PPM1A OE but significantly decreased in study cells co-transfected with both miR-487a-3p and PPM1A OE plasmid (Figure 5(b)). Next, cell viability studies through CCK-8 showed a significantly reduced proliferation following miR-487a-3p + PPM1A OE co-transfection compared to PPM1A OE (Figure 5(c,d)). Later, cell growth assessment was undertaken on the CAL-27 and TCA8113 transfected with a control plasmid, PPM1A OE, and miR-487a-3p + PPM1A OE. Colony formation assay findings showed increased growth in TCA8113, and CAL-27 cells transfected with PPM1A OE but significantly decreased when TCA8113 and CAL-27 co-transfected with miR-487a-3p and PPM1A OE plasmid (Figure 5(e)). Finally, transwell experiments also showed an increased cell invasion in TCA8113, and CAL-27 cells transfected using PPM1A OE but a significant decrease in TCA8113 and CAL-27 co-transfected with a miR-487a-3p and pcDNA-PPM1A plasmid (Figure 5(d)). Overall our observations indicate that overexpression of PPM1A exerts oncogenic effects, but mimicking miR-487a-3p exerts anti-oncogenic impacts in oral squamous cell carcinoma cells by alleviating the cell growth and cell invasion.

(a) Expression of miR-487a-3p in OSCC cells under transfection of miR-487a-3p and PPM1A-OE plasmid. (b) Expression of PPM1A protein in OSCC cells under transfection of miR-487a-3p and PPM1A-OE plasmid. (c,d) Cell proliferation in OSCC tissue under transfection of miR-487a-3p and PPM1A-OE plasmid. (e) Colony formation analysis for cell growth under transfection of miR-487a-3p and PPM1A-OE plasmid. (f) Transwell invasion analysis for OSCC cell invasion under transfection of miR-487a-3p and PPM1A-OE plasmid. *P < 0.05 and **P < 0.01.

## 4. Discussion

OSCC remains among the most malignant cancers, and its incidence is continuously rising in developing countries [20]. Despite OSCC treatment progress, low survival rates warrant more attention on newer targeted therapies. Gene therapy, being among the new therapeutic approaches, has led to tremendous results [21]. As among the gene therapy approach, the current study mainly delved into miR-487a-3p and PPM1A role on OSCC. The following conclusion confirmed miR-487a-3p and PPM1A inhibitory role on proliferation and migration of OSCC through PPM1A targeting.

Previous studies provided briefs on a miR-487a-3p role as a neoplasm repressor on various kinds of malignancies. Zhou et al. studied miR-487a-3p function against pancreatic cancer [22], and its overexpression remarkably reduced pancreatic cancer cells’ migration and proliferation. The study also reported that miR-487a-3p target SMAD7, which is the downstream signal conveyed in pancreatic cancer. Consequently, overexpression of SMAD7 is reversed by the suppressor effects of miR-487a-3. Similarly, Wang et al. reported the role of miR-487a-3p in prostate cancers [23], whereby miR-487a-3p over-expression repressed Prostrate Cancer cell growth, invasion, and migration via targeting CCND1.

To further study the importance of miR-487a-3p in OSCC, the present experiment’s RT-qPCR findings confirmed its downregulation in OSCC cells. miR-487a-3p expression was also reduced in various human malignancy types, such as prostate cancers [23]. According to the present study’s data analysis, miR-487a-3p reduced the proliferation, migration, and invasion while enhancing OSCC cell apoptosis.

In elucidating the mechanisms, this study hypothesized that miR-487a-3p regulated the biological ability of OSCC via controlling corresponding protein genes. A recent study has distinguished genes like CTLA4, MARCH5, and FOXO3 as the targets of miR-487a-3p [24]. Investigations have also clarified the essential function of PPM1A in tumor repression. According to Zhang et al., PPM1A negatively modulates various signaling pathways essential for invasion and migration of trophoblast [19]. PPM1A is also regulated by miR-135b in promoting proliferation and invasion of osteosarcoma cells [25]. We consequently aimed to study the association between PPM1A and miR-487a-3p and prove the involvement of PPM1A in OSCC progression. RT-qPCR findings confirmed the high expression of PPM1A in OSCC cells.

To confirm the interaction between PPM1A and miR-487a-3p, dual-luciferase reporter and western blot assays affirmed PPM1A 3’UTR-targeting by miR-487a-3p, thus downregulating its expression. To further clarify any possible critical tumor suppression miR-487a-3p function through PPM1A expression modulation, CAL-27 and TCA8113 cells were transfected using miR-487a-3p mimics and PPM1A OE plasmid. In agreement with the postulated hypothesis, miR-487a-3p mimics and PPM1A OE co-transfection increased cell growth and cell invasion in OSCC.

In brief, the current investigation provides a noble perspective of the association between miR-487a-3p and PPM1A in OSCC. The miR-487a-3p overexpression inhibits OSCC cell growth and invasion, miR-487a-3p targets the PPM1A gene in OSCC, and the miR-487a-3p overexpression may rescue the oncogenic effects of PPM1A on oral squamous cell carcinoma cells. In conclusion, this investigation firstly confirmed that miR-487-3p accelerated cell apoptosis and inhibited invasion and migration of OSCC by the direct regulation of PPM1A. The study offered the in vitro evidence to properly understand the increase of miR-487a-3p or the decrease of PPM1A as a tumor repressor in OSCC. Besides, miR-487a-3p/PPM1A targeting could be a possible therapeutic approach for the treatment of OSCC.

## Research highlights

- Expression of miR-487a-3p is downregulated in oral squamous cell carcinoma (OSCC) tumors and cell lines
- Higher expression of PPM1A is associated with increase cell growth and invasiveness of OSCC cell lines
- Mir-487a-3p directly binds with the 3’UTR of PPM1A and inhibits its expression
- Exogenous delivery of miR-487a-3p leads to the downregulation of PPM1A expression and inhibits the growth and invasiveness of OSCC.
